# Defining emergency department episodes by severity and intensity: A 15-year study of Medicare beneficiaries

**DOI:** 10.1186/1472-6963-10-173

**Published:** 2010-06-21

**Authors:** Brian Kaskie, Maksym Obrizan, Elizabeth A Cook, Michael P Jones, Li Liu, Suzanne Bentler, Robert B Wallace, John F Geweke, Kara B Wright, Elizabeth A Chrischilles, Claire E Pavlik, Robert L Ohsfeldt, Gary E Rosenthal, Fredric D Wolinsky,

**Affiliations:** 1 Department of Health Management and Policy, College of Public Health, the University of Iowa, Iowa City, Iowa, USA; 2 Kyiv School of Economics, and Kyiv Economics Institute, Kyiv, Ukraine; 3 Department of Epidemiology, College of Public Health, the University of Iowa, Iowa City, Iowa, USA; 4 Center for Research on the Implementation of Innovative Strategies into Practice, Iowa City Veterans Administration Medical Center, Iowa City, Iowa, USA; 5 Department of Biostatistics, College of Public Health, the University of Iowa, Iowa City, Iowa, USA; 6 Department of Economics, Tippie College of Business, the University of Iowa, Iowa City, Iowa, USA; 7 Department of Geography, College of Liberal Arts and Sciences, the University of Iowa, Iowa City, Iowa, USA; 8 Department of Health Management and Policy, School of Rural Public Health, Texas A&M University Health Science Center, College Station, Texas, USA; 9 Department of Internal Medicine, Carver College of Medicine, the University of Iowa, Iowa City, Iowa, USA

## Abstract

**Background:**

Episodes of Emergency Department (ED) service use among older adults previously have not been constructed, or evaluated as multi-dimensional phenomena. In this study, we constructed episodes of ED service use among a cohort of older adults over a 15-year observation period, measured the episodes by severity and intensity, and compared these measures in predicting subsequent hospitalization.

**Methods:**

We conducted a secondary analysis of the prospective cohort study entitled the Survey on Assets and Health Dynamics among the Oldest Old (AHEAD). Baseline (1993) data on 5,511 self-respondents ≥70 years old were linked to their Medicare claims for 1991-2005. Claims then were organized into episodes of ED care according to Medicare guidelines. The severity of ED episodes was measured with a modified-NYU algorithm using ICD9-CM diagnoses, and the intensity of the episodes was measured using CPT codes. Measures were evaluated against subsequent hospitalization to estimate comparative predictive validity.

**Results:**

Over 15 years, three-fourths (4,171) of the 5,511 AHEAD participants had at least 1 ED episode, with a mean of 4.5 episodes. Cross-classification indicated the modified-NYU severity measure and the CPT-based intensity measure captured different aspects of ED episodes (kappa = 0.18). While both measures were significant independent predictors of hospital admission from ED episodes, the CPT measure had substantially higher predictive validity than the modified-NYU measure (AORs 5.70 vs. 3.31; p < .001).

**Conclusions:**

We demonstrated an innovative approach for how claims data can be used to construct episodes of ED care among a sample of older adults. We also determined that the modified-NYU measure of severity and the CPT measure of intensity tap different aspects of ED episodes, and that both measures were predictive of subsequent hospitalization.

## 

Emergency department (ED) use in the U.S. has increased substantially. Between 1993 and 2003, the US population grew by 12% and hospital admissions increased by 13%, but ED visits went up by 26% [1]. This increase in ED use occurred simultaneously with a net loss of 703 hospitals (11%) and a net decline of 425 EDs (9%) [2]. In 2006 the Institute of Medicine (IOM) published its landmark Report on the Future of Emergency Care, and emphasized that the "increasing use of the emergency care system...represents failures of the larger health care system--the growing numbers of uninsured Americans, the limited alternatives available in many communities, and the inadequate preventive care and chronic care received by many" [3]. The IOM specifically contended the market for ED services was not operating at equilibrium, and suggested the lack of equilibrium created several substantive public health problems. Overcrowding was considered among the most serious of these, as more than 90% of EDs reported overcrowding is a serious problem [4] and 40% reported that the problem occurs daily [5].

Our goal was to advance the empirical investigation of ED use in two ways. We first applied an innovative method to construct episodes of ED care. This advances the field because methods employed in previous research [6-9] may have overestimated the extent of ED use. In particular, investigators have equated the number of ED claims with the number of ED visits [10], and we determined this approach to be suboptimal because it does not acknowledge that multiple claims can be submitted during a single ED visit [11]. For example, during a single ED episode, a patient may be seen by two different physicians, each of whom (if self-employed) generate separate claims for the patient encounter while the hospital outpatient department also generates a bill for the technical component of care provided in the ED. Thus, three claims are submitted for a single episode of ED care. The extent to which the single claim equals a single visit approach overestimates ED use has never been evaluated against an approach in which claims are bundled into single episodes of care. Second, given the complexity of ED encounters [12,13], we assumed ED episodes are multi-dimensional phenomena and different measures might be needed to illuminate the distinct aspects of the episodes. This advances previous work in which ED use was measured along a single dimension of clinical severity.

In this study, episodes of ED care were identified using data from a nationally representative cohort of older adults. While older adults certainly do not represent all ED service users, focusing on them is warranted because persons over 65 figure so prominently into the ED crisis. Older adults have higher ED use rates, more urgent visits, longer stays, greater likelihoods of hospital admission, higher repeat ED use, and worse health outcomes than their younger counterparts. The role of older adults in the ED crisis also is likely to increase as the population ages. In the coming decade persons over 65 may have a disproportionate impact on ED overcrowding, posing threats to both the health care safety net and the quality of patient care [14-16].

We described episodes of ED care using two distinct measures. We measured clinical severity with a modified NYU-algorithm that used diagnostic information to categorize ED visits along a continuum reflecting the urgency of required care [17]. CPT codes were used to measure the intensity of the ED episode. In particular, evaluation and management codes 99281 to 99285 provided an indication of the services delivered and staff time expended during an ED episode [18]. While clinical severity of ED care has been measured previously, it has not been applied to episodes of ED care among a population-based sample of older adults, nor compared to another measure capturing the intensity of the ED episode. Finally, we estimated the predictive validity of these two measures by using subsequent hospitalization from the ED as a primary outcome of the ED episode. Determining how well these different measurements predict subsequent hospitalization helps illuminate a critical pathway of health service use travelled by many older adults [11].

## Methods

We conducted a secondary analysis of the prospective cohort study known as the Survey on Assets and Health Dynamics among the Oldest Old [AHEAD; 19, 20]. AHEAD is a national, omnibus health and retirement longitudinal data source of Medicare-eligible older adults administered by the Survey Research Center at the University of Michigan. AHEAD is a prospective cohort study in which subject interviews have been conducted about every two years since 1993. The survey questions field a wide array of information including: demographics, cognitive performance, physical and functional health, Medicaid eligibility, family structure, care-giving, and out-of-pocket costs for health and social services. Human subjects approval for our study was provided by the AHEAD Restricted Data Use Committee (# 2003-006), the University of Iowa IRB (# 2003-03008), and the Centers for Medicare and Medicaid Services (Data Use Agreement # 14807). Two sampling frames were used in creating AHEAD--a 1992 multi-stage household screening process, and a supplemental sample of persons ≥80 years old from the Medicare Master Enrollment File. Baseline interviews were conducted in 1993 with 7,447 participants ≥70 years old (response rate = 80.4%).

### Sample

We created our analytic sample by linking baseline AHEAD interviews to Medicare inpatient, outpatient, and carrier claims for calendar years 1991-2005. Among the 7,447 older adults who completed the baseline AHEAD interviews in 1993 and 1994, we excluded 802 from this study because their AHEAD data could not be linked to their Medicare claims. We also excluded 604 participants enrolled in managed Medicare during the two years prior to baseline because these plans did not provide comparable data to the fee-for-service Medicare plans in which all AHEAD participants were enrolled. The 530 AHEAD participants who required proxy respondents at the baseline interview also were excluded because they did not complete cognitive and psychosocial protocols that measured risk factors included in our analysis. In this study, the number of AHEAD participants with linked Medicare claims data totaled 5,511 men and women (74.0% of the original AHEAD sample). In previous work [21], we developed propensity scores to address the potential sample selection bias based on our exclusion criteria but found that such adjustments did not have a substantial impact on the result. In this study, we could not use these adjustments because our unit of analysis was the ED episode rather than the individual. Nonetheless, based on our previous work, we assumed the bias would be minimal.

### Medicare Claims

Three Medicare standard analytic files contain data on the provision of care in the ED--the outpatient claims files, the carrier claims files, and the inpatient claims files. ED services provided by physicians employed as hospital staff are submitted by the hospital as outpatient claims, and these claims reflect both the professional (i.e., physician effort) and technical (i.e., lab testing) components of ED care. Physicians who are either self-employed or part of a larger, hospital affiliated physician group submit their ED claims to a designated Medicare carrier. Therefore, ED claims in either the outpatient or carrier files are easily identified with CPT evaluation and management codes 99281-99285. This approach to identifying ED claims previously was used by the IOM, and accounts for over 80% of all Medicare expenditures for ED services [3].

We also could have identified visits to the ED with inpatient claims and from other outpatient and carrier claims that did not include CPT codes 99281-99285. For example, 5,854 ED claims for our study sample were uniquely found in the inpatient claims files (i.e., they did not have a corresponding outpatient or carrier claim with a CPT code 99281-99285). This occurred because Medicare statutes dictate that when a patient who presents to the ED subsequently is admitted to the hospital, the services provided in the ED must be "rolled" into the inpatient claim under bill values of 111-119 or revenue center codes (RCCs) of 0450-0459. We also could have used claims with CPT codes 99291 and 99292 to identify ED care that was provided to older persons who were critically ill or critically injured [22]. Our initial review of the sample data revealed these two codes appeared on 1,872 separate claims. However, we chose not use any of these claims to construct additional episodes of ED care because the episodes likely included services provided outside of an ED in locations such as a coronary care unit, intensive care unit, or respiratory care unit. This did not necessarily reflect a comparable construct of ED use. Moreover, these claims did not allow us to construct a measure of service intensity comparable to the information provided by CPT codes 99281-99285 in the outpatient and carrier claims.

### ED Episodes

In the absence of prior work to define ED episodes from Medicare claims [6-10], we developed a bundling algorithm reflecting Medicare billing policy to identify ED episodes. For the outpatient files, we bundled claims for which the "from" and "through" dates overlapped or were within 3 days, consistent with Medicare policy requiring outpatient claims to be bundled if they occur within 72 hours [22,23]. For the carrier files, we bundled claims with overlapping dates or those that were within 1 day of each other. This was necessary because Medicare claims have date but not time stamps, and therefore it is possible for a late-night ED encounter to carry over into the next calendar day. We then bundled the outpatient and carrier claims with overlapping dates and defined them as belonging to the same ED episode. We recognized that bundling claims over a consecutive three-day period may underestimate the actual number of episodes given that some individuals may enter and complete an ED episode on one day and then return to the ED on the next day. Therefore, we identified the number of episodes in which all claims were filed in a one day period from those in which claims spanned a two or three day period.

### Measures of ED Episodes

We used two approaches to measure each ED episode in terms of severity and intensity. Our first approach relied on a modified-NYU algorithm. Originally, Billings et al. [24] created an algorithm (i.e., the NYU algorithm) to classify the severity of ED care by using the ICD9-CM diagnostic codes as identified in the ED. Using the diagnostic information, Billings and his colleagues calculated the probability that an ED claim fell into one of four categories: 1) non-emergent (NE); 2) emergent, primary care treatable (EPCT); 3) ED care needed, preventable/avoidable (EDCNPA); and 4) ED care needed, not preventable avoidable (EDCNNPA; http://wagner.nyu.edu/chpsr/index.html).

NE cases are those in which the patient's initial complaint, presenting symptoms, vital signs, medical history, and age indicated that immediate medical care was not required within 12 hours. The EPCT cases are those in which emergent care was required within 12 hours, though the presenting problem did not require continuous observation and no procedures were performed or resources used (i.e., a CT scan or lab work) that were not available in a primary care setting. The EDCNPA cases indicate that emergency department care was required, but the emergent nature of the condition was potentially preventable/avoidable if timely and effective ambulatory care had been received. Finally, EDCNNPA cases are those in which emergency department care was required and ambulatory care treatment could not have prevented the condition.

Since administrative records do not contain adequate information to make absolute determinations as to the appropriate category, the original NYU algorithm assigns probabilities that a visit falls into each of the four above categories, yielding four probability estimates. In developing this algorithm, Billings et al. did not classify visits to the ED that involved trauma, alcohol, drug-related, or psychiatric diagnoses. They also did not categorize bladder and urinary tract infections, and other infrequent diagnostic conditions.

Wharam et al. [17] simplified Billings et al.'s approach by generating a single measure of ED severity based on the summation of probabilities. Their modified-NYU algorithm, which we used in this study, defined a severe visit (modified-NYU rating = 3) as one in which the probability that the ED was needed was ≥75% (e.g, EDCNNPA + EDCNPA ≥0.75). ED episodes were defined as non-severe (modified-NYU rating = 1) if the probability that ED care was needed was ≤ 25% (i.e. EDCNNPA + EDCNPA ≤ 0.25). ED episodes that did not meet either criteria were defined as indeterminate severity (modified-NYU rating = 2).

Our second measurement relied on the actual CPT codes. As mentioned earlier, all physician claims for professional services provided in the ED receive one of five CPT codes [18], which reflect the intensity of services provided to the patient. The lowest CPT code (i.e., 99281) indicates that a minimal number of services were provided, services that could have been provided in alternative settings (i.e., physician offices or other outpatient settings). The highest code (i.e., 99285) reflects the amount of care provided during a severe or life-threatening emergency situation. In this study, claims were classified as highest intensity if they received a 99285 code; claims receiving 99281-99284 codes were classified as lower intensity.

**Figure 1:**
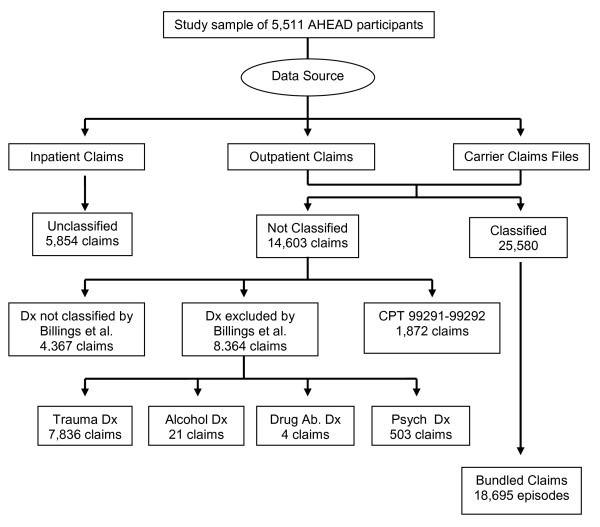
**Source and distribution of ED claims excluded and included in analysis**.

We initially identified 38,311 claims for ED visits among our study sample. However, 12,731(33.2%) of these included diagnoses of trauma (n = 7,836), alcohol (n = 21), drug-related (n = 4), psychiatric diagnoses (n = 503), and 4,367 other diagnoses that were not classifiable using the modified-NYU algorithm. As such, these claims were removed from further analysis. Moreover, as we bundled the remaining claims into episodes and measured the severity and intensity of each ED episode, we occasionally identified more than one modified-NYU or CPT code within a single episode. This was not problematic when those multiple codes were concordant, which was the case for 96% of the episodes with multiple modified-NYU codes, and for 82% of the episodes for which there were multiple CPT codes. When the multiple modified-NYU codes or CPT codes were not concordant, we selected the codes reflecting the highest level of severity or intensity to define the episode. Figure 1 presents the data sources and corresponding number of ED claims excluded and included in our analysis.

### Analysis

We plotted the annual trends in the per capita number of ED episodes among the study sample from 1991 to 2005, and examined trends pertaining to the two measures of ED episodes (i.e., modified-NYU severity & CPT intensity) and tested for their concordance over the 15-year observation period. Then, following on work completed by Ballard et al. [25], we observed which classification approach had greater predictive validity relative to being hospitalized. Hospitalization following the ED episode (i.e., admission from the ED) was defined as a binary outcome and regressed onto the modified-NYU code 3 (using 1 & 2 as the pooled reference) and CPT code 99285 (using 99281-99284 as the pooled reference).

We then regressed hospitalization onto a model that included several other risk factors. Because 4,171 (76%) of the 5,511 AHEAD participants had ≥1 ED episode, and these ED episodes were clustered, we incorporated generalized estimating equation (GEE) methods using an exchangeable correlation structure [26]. Several model development strategies were employed, including forced entry of all potential risk factors, as well as forward, backward, and stepwise selection. All risk factors statistically significant in one or more of those approaches were retained in the final model.

Our initial variable selection for the model was consistent with Andersen's widely used behavioral model of health services use [27]. We chose age, race, sex, marital status, living alone, and the importance of religion as demographic factors. Socioeconomic characteristics included education, income, and the number of health insurance policies. Health behaviors were represented by smoking status, alcohol use, and body mass index. Functional status assessment included a 5-item activity of daily living (ADL) index, a 5-item instrumental ADL (IADL) index, a single self-reported health question, and an 8-item version of the Center for Epidemiologic Studies Depression rating scale [CESD-8; [28]]. Cognitive assessment was evaluated using the Telephone Interview to Assess Cognitive Status [TICS; [29]], as well as delayed word recall. Disease history was tapped by self-reports of having been told by a physician that one has angina, arthritis, cancer, diabetes, heart attack, heart disease, stroke, hip fracture, hypertension, lung disease, or a psychiatric condition. Geographic factors included population density, Census region, the number of hospital beds and physicians per 1,000 persons in the county of residence (imported from the Area Resource File and based on the participant's place of residence at baseline), and perceived neighborhood safety. Health services use included the number of visits to a physician and hospital episodes in the previous year.

Because the preponderance of these risk factors for hospitalization were measured at baseline, we created an additional measure which captured the change in an individual's self-rated health from the baseline interview to the interview that preceded the first ED episode. We sorted the measures of change in self rated health into four groups: those individuals with no change in self rated health from the baseline to the interview prior to the first ED episode (780 out of 4,171 respondents); those with decline in self reported health (676 respondents); those with increases in self reported health (408 respondents), and a group in which only one assessment of self-rated health was provided at baseline (2,307 out of 4,171). To capture secular trends and access differentials we also used Medicare claims to account for when the ED episode occurred during our analytic period, and whether it occurred on a weekend or holiday.

## Results

**Figure 2:**
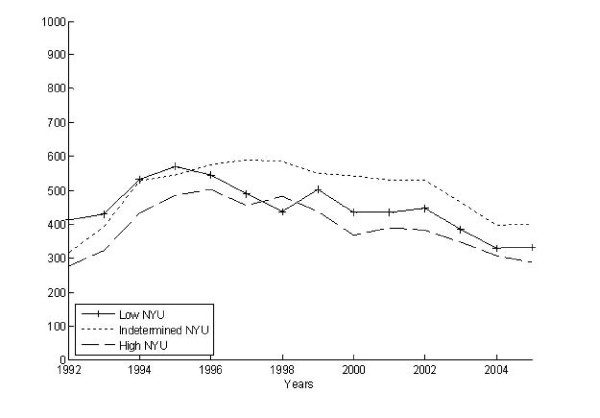
**Modified-NYU severity over time for all ED episodes**.

**Figure 3:**
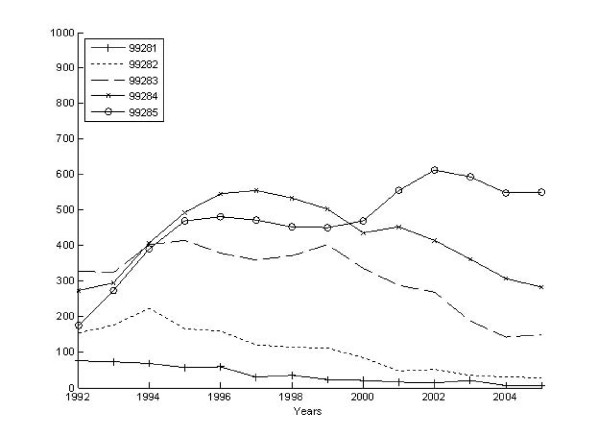
**CPT intensity over time for all ED episodes**.

We identified 25,580 claims associated with an ED visit and then bundled these into 18,695 episodes of ED care. Three-fourths (4,171) of the 5,511 AHEAD participants had at least 1 ED episode during the observation period, and the per capita number of ED episodes ranged from 0 to 10, with a mean of 4.5 episodes among those having one or more. The volume of ED visits rose slightly over time from 1,451 episodes per 1,000 participants in 1992 to 1,688 episodes per 1000 participants in 2005. In part, this reflects the aging of our cohort, inasmuch as the mean age of those having ED episodes increased steadily from 78 to 88 years old during this same period. Figures 2 and 3 illustrate the differing patterns in the number of ED episodes measured as the highest severity and intensity over the 15 year period. At the start of the observation period, the most severe and intense ED episodes were less common than all other types. However, from 1992 to 2005, the proportion of the highest severity episodes increased slightly whereas the proportion of the highest intensity episodes exploded. By the end of the observation period, severe episodes remained less common than indeterminate and low severity episodes while nearly 50% of all ED episodes were being classified as high intensity.

Table 1 shows the number of claims per ED episode by source (i.e., from the outpatient or carrier files). Of the 18,695 ED episodes, 11,151 (59.6%) included only one carrier claim, 1,241 (6.6%) included only one outpatient claim, and 5,432 (29.1%) included one outpatient and one carrier claim. These patterns accounted for 95.3% of the ED episodes. Further, only 502 (2.7%) of the ED episodes were constructed with claims that spanned three or more days.

**Table 1:** Claims Source for All ED Episodes 1991-2005 (n = 18695)

	Number of institutional outpatient claims					
Number of carrier claims	Count	0	1	2	3 or more	Total
	
	0	0	1241	18	4	1263
	
	1	11151	5432	120	6	16709
	
	2	397	183	10	0	590
	
	3 or more	99	33	1	0	133
	
	Total	11647	6889	149	10	18695

Table 2 presents a cross-tabulation of modified-NYU coded episodes by CPT episodes. One difference is the number of episodes rated high severity by the modified-NYU measure (5,473; 29%) compared to the number rated as the highest intensity by the CPT measure (6,492; 35%). Moreover, the 6,492 episodes defined as severe using the CPT 99285 code are split between modified-NYU severity rating 1 (19%), modified NYU severity rating 2 (41%), and modified-NYU severity rating 3 (41%). If the measures agreed well, we would expect a disproportionately larger share of these 99285 codes to be rated a 3 on the modified-NYU severity measure. To verify these observed differences, we cross-classified the binary severity measures (modified-NYU code 3 and CPT code 99285); the resulting kappa statistic of 0.18 reflected the substantial lack of agreement between the two measures (95% CI; 0.167 - 0.196).

**Table 2:** Concordance of CPT codes By Modified-NYU Codes

	Modified-NYU Severity Rating			Total
CPT code	1	2	3	

99281	323 (65%)	110 (22%)	67 (13%)	500

99282	936 (62%)	386 (26%)	178 (12%)	1500

99283	2067 (48%)	1437 (33%)	842 (19%)	4346

99284	1752 (30%)	2362 (40%)	1743 (30%)	5857

99285	1206 (19%)	2643 (41%)	2643 (41%)	6492

Total	6284	6938	5473	18695

**Figure 4:**
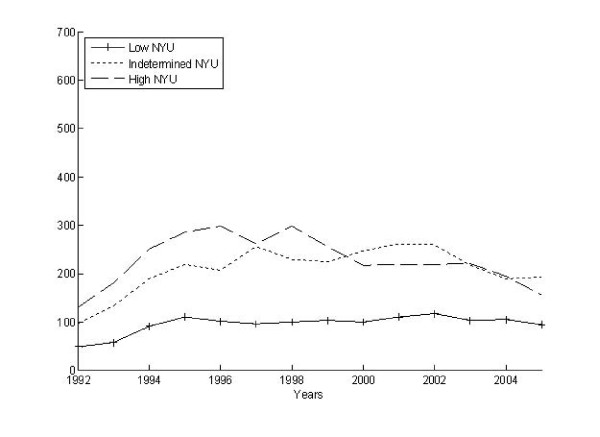
**Modified-NYU severity over time for ED episodes that resulted in hospitalization**.

**Figure 5:**
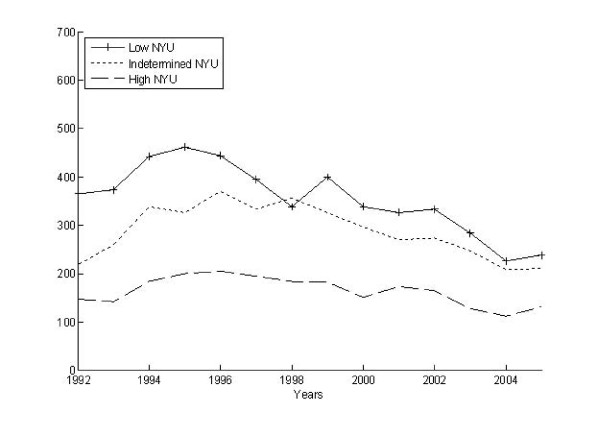
**Modified-NYU severity over time for ED episodes that did not result in hospitalization**.

**Figure 6:**
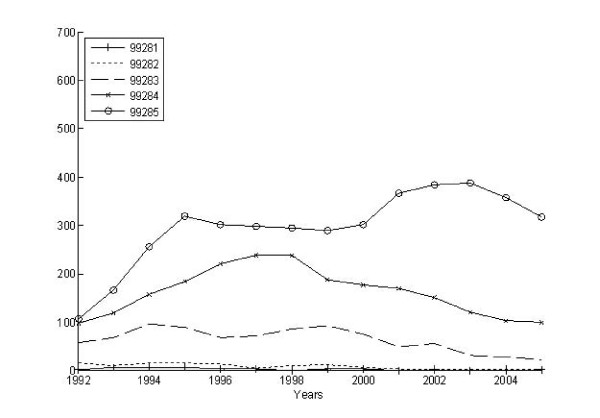
**CPT intensity over time for ED episodes that resulted in hospitalization**.

**Figure 7:**
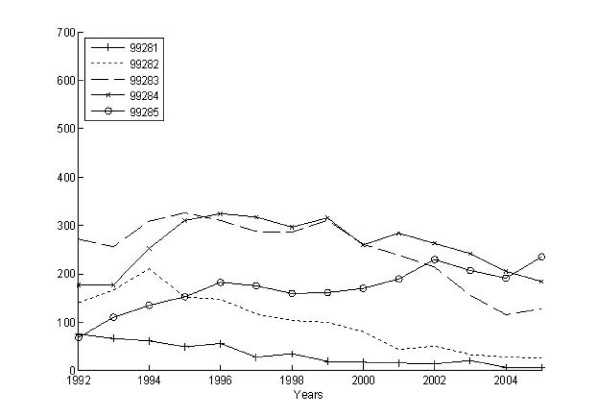
**CPT intensity over time for ED episodes that did not result in hospitalization**.

Figures 4 and 5 present data on hospitalization as an outcome of the ED episode by levels of severity. Figures 6 and 7 do the same by levels of intensity. Overall, 40% of the ED episodes resulted in hospitalization (i.e., admission to the hospital directly from the ED visit). The column totals in Table 3 illustrate the proportion of hospitalizations coded with each of the modified-NYU severity ratings. The relationship between hospitalization and ED episode severity indicates 21.2% of those rated as 1 or "not severe" were hospitalized, 42.0% of those rated 2 or "indeterminate severity" were hospitalized, and 58.1% among those episodes rated as a 3 or "high severity" were hospitalized. In an effort to optimize the sensitivity of the NYU measure, we recalibrated the measure first as quartiles and then as quintiles. This did not substantially change the relationships between measures of ED severity and subsequent hospitalization. Alternatively, the row totals in Table 3 illustrate the proportion of these hospitalizations coded with each CPT code (99281-99285). The proportion of CPT codes 99281 and 99282 that resulted in hospitalization were low (6.8% & 7.3%) compared to the proportion of 99283 (20.3%), 99284 (38.6%) and 99285 (63.7%) codes that resulted in hospitalization. ANOVAs for the proportion hospitalized by the categorical measures of severity and intensity indicated significant differences at the p < 0.001 level.

**Table 3:** CPT Intensity Codes by NYU Severity Ratings (and Rates of Hospitalization)

	Modified NYU Rating 1	Modified NYU Rating 2	Modified NYU Rating 3	Total
99281	323 (2.5%)	110 (9.1%)	67 (23.9%)	500 (6.8%)

99282	936 (2.9%)	386 (9.1%)	178 (26.4%)	1500 (7.3%)

99283	2067 (9.6%)	1437 (23.0%)	842 (41.9%)	4346 (20.3%)

99284	1752 (24.9%)	2362 (37.2%)	1743 (54.2%)	5857 (38.6%)

99285	1206 (54.7%)	2643 (62.8%)	2643 (68.8%)	6492 (63.7%)

Total	6284 (21.2%)	6938 (42.0%)	5473 (58.1%)	18695 (39.7%)

To compare the predictive validity of the two measurement approaches, we first estimated crude odds ratios for the measures of severity and intensity, and then estimated adjusted odds ratios to identify their independent effects. Both binary severity classification markers--modified-NYU and CPT--had statistically significant effects (ORs of 2.32 & 4.26; p < .001). We then estimated a multivariable logistic regression model using GEE methods. The final model selected several covariates including age, race, status as a current smoker, geographic location and time period in which the ED episode occurred, and after adjusting for these, the modified-NYU code 3 measure had an adjusted odds ratio AOR of 3.31 (p < .0001), and the CPT code 99285 had an AOR of 5.70 (p < .0001). We also included an interaction term and found that having both a high level of clinical severity (NYU 3) and intensity (CPT 5) increased the odds for being hospitalized (AOR 8.09; p < .0001).

## Discussion

This study analyzed ED episodes using a large, nationally representative sample of Medicare beneficiaries with data reflecting a variety of individual characteristics linked with Medicare claims for calendar years 1991-2005. There are few studies that can provide such considerable external validity. In this particular study, we created a definition for the episodes of care provided within EDs, and found our definition improved upon previous efforts in which ED use was defined as separate visits created from individual claims. In our approach, in which separate claims were bundled into ED service episodes, we observed a substantial difference between the total number of ED episodes and ED visits as they would have been defined in previous research. This finding suggests that the application of the one claim equals one visit approach may inadvertently inflate the frequency of this particular service outcome and produce biased estimates.

We also observed a significant lack of agreement in the two measures of ED episodes, and it appears we have identified two distinct, time-varying constructs to characterize episodes of ED care. This was to be expected, to some extent, given the two different methods of measurement; but it appears we may have identified two distinct constructs that are differentially sensitive to characterizing changes in ED care episodes over time.

Moreover, while both measures predicted subsequent hospitalization, the CPT classification approach demonstrated substantially greater predictive validity than the modified-NYU approach. While the NYU approach has been shown to be predictive of subsequent hospitalization [25], we are reminded that the original algorithm was developed solely on ED visits that did not result in hospitalization [30]. Indeed the original NYU algorithm was developed to aid policymakers in evaluating access to care in a community, and the measure of ED utilization served as a "window" to the status of the safety net. So it should not be so surprising that it is not as sensitive to predicting hospitalization. In contrast, the CPT codes may be a more valid predictive measure because they capture more subtle elements that correspond with a need for hospitalization, and these elements may not be adequately captured using the ICD9-CM codes featured in the modified-NYU measure. The CPT coding also captures the more obvious elements of an intense episode that lead to hospitalization such as the need for specialty physician consultation, multiple systems evaluation, and the time staff spent observing an individual in the ED.

We may find these measures differentially useful in predicting other outcomes such as continuity of care, nursing home placement, or death, or that they may vary if other populations are considered. In this study, however, we resolved that understanding more about the pathway between the ED and the hospital would be particularly critical given the expanding role that the aging population is having on the use of both EDs and inpatient hospital services. In fact, we found the link between ED care and inpatient hospital admissions presented a high amount of face validity in regard to depicting service use pathways as many older adults in our sample were admitted to the hospital whereas very few died subsequent to an ED episode. We also found this approach to be consistent with other research considering the predictive utility of distinguishing between non emergent and emergent care and subsequent hospitalization [25].

We recognize that there are limitations to this study. We excluded data provided by the AHEAD participants who were not self-respondents and those who were enrolled in Medicare managed care at baseline. While these exclusions may have introduced some selection bias, the direction of that bias remains unknown. We also may have erred in bundling the claims into unique ED episodes since the Medicare claims contain date but not time stamps. However, the potential lack of accuracy was considered to be minimal. Further, when forced to bundle two different claims into an ED episode, we may have over-estimated the number of the most severe ED episodes because we always selected the highest assigned CPT or modified-NYU code rating. This too is not likely to have been much of a problem because most multiple CPT (82%) or multiple modified-NYU codes (96%) were intensity- or severity-concordant.

The most substantive limitation of this effort was the inability to incorporate all of the claims for ED service use among the AHEAD sample. In developing our method of constructing ED episodes, we omitted inpatient claims, claims with select CPT codes, and those associated with particular diagnostic conditions (e.g., trauma, drug abuse), and all other claims associated with the provision of care within the ED. Given the large number of these claims, their omission certainly warrants further consideration. Two questions come to mind. How exactly were the episodes of care for individuals who presented in the ED and then immediately admitted to inpatient care different in terms of severity and intensity of care provided, or some other characteristic? How does the exclusion of traumatic and other particular types of ED episodes (e.g., substance abuse, psychiatric) limit our understanding of the ED crisis?

## Conclusion

The objectives of this study were to accurately define ED episodes of care among a cohort of older adults, to measure the severity and intensity of these ED episodes using two distinct approaches, and to compare the validity of these two measurement approaches using admission to the hospital from the ED as the outcome of primary interest. We demonstrated that defining ED visits as episodes of care using an administrative dataset is relatively straightforward and results in a valid depiction of service use. We also identified a measure of ED episodes that is strongly predictive of hospitalization and may serve as a valuable tool for monitoring the well traveled pathway between the ED and the hospital among older adults [31]. Besides addressing methodological shortcomings in previous work, our effort to define ED care as multi-dimensional episodes responds to the increasing interest among policy makers and payers to move away from a system in which a fee is paid for each service claimed toward a system in which payment is defined by clinical severity and the intensity of patient care provided during a discrete period of time [32,33].

## Competing interests

The authors declare that they have no competing interests.

## Authors' contributions

FDW conceived of the study, wrote the grant application, designed the analyses, interpreted the results, and revised the manuscript. EAC, JFG, RLO, GER, and RBW participated in the conceptualization of the grant application and the overall study design, and provided their expertise throughout the study. MO and EAC constructed episodes of ED use, constructed measures of severity and intensity, completed statistical analyses, and prepared the manuscript. SEB, LL & MO assisted with analyses. BK contributed to design study, assisted with statistical analyses, interpreted the results, and prepared the manuscript. MO, EAC & KBW assisted with all data linkage, merging, and recoding. CEP harvested and prepared all of the geocoded information from Census and other sources for migration and linkage. All authors participated in numerous meetings to outline, read, critique, revise, re-read, and grant approval the final manuscript. FDW, BK, MO, and EAC had full access to all of the data in the study and take responsibility for the integrity of the data and the accuracy of the data analysis. All authors read and approved the final manuscript.

## Pre-publication history

The pre-publication history for this paper can be accessed here:

http://www.biomedcentral.com/1472-6963/10/173/prepub
